# Roundup^®^ Original DI promotes proliferation in papillary thyroid carcinoma cells: a comparative study highlighting cell-type-dependent effects

**DOI:** 10.3389/fendo.2026.1745247

**Published:** 2026-04-23

**Authors:** Izabela Fernanda Dal’ Bó, Natássia Elena Bufalo, Valdemar Máximo, Laura Sterian Ward

**Affiliations:** 1 Laboratory of Cancer Molecular Genetics, School of Medical Sciences, University of Campinas (UNICAMP), Campinas, SP, Brazil; 2 Department of Medicine, São Leopoldo Mandic and Research Center, Campinas, SP, Brazil; 3 Max-Planck University Center (UniMAX), Indaiatuba, SP, Brazil; 4 Institute for Research and Innovation in Health (i3S), University of Porto, Porto, Portugal; 5 Institute of Molecular Pathology and Immunology of the University of Porto (Ipatimup), Porto, Portugal; 6 Department of Pathology, Faculty of Medicine of the University of Porto (FMUP), Porto, Portugal

**Keywords:** cytotoxicity, endocrine disruptor, glyphosate, pesticide, proliferation, thyroid

## Abstract

**Introduction:**

Glyphosate-based herbicides (GBHs), such as Roundup®, are ubiquitous environmental contaminants with emerging endocrine-disrupting properties. Based on previous findings of non-monotonic effects in thyroid models, this study investigated lineage-dependent cytotoxic and proliferative responses to Roundup® in thyroid cells with different cytogenic profiles.

**Methods:**

Human papillary carcinoma (BCPAP, BRAFV600E) and anaplastic carcinoma (8505C) cells were exposed to Roundup® Original DI (6.5–6500 µg/L) for 24 and 48 hours. Cell viability was assessed using trypan blue exclusion and CCK-8 assays, and proliferation was measured by BrdU incorporation. The data were integrated with previous results from normal (Nthy-ori 3-1) and papillary (TPC-1) cell lines for comparative analysis.

**Results:**

Exposure to Roundup® induced specific effects for each cell type. The BCPAP cell line demonstrated significant sensitivity, with reduced viability and sustained proliferative stimulation at low concentrations. In contrast, anaplastic 8505C cells proved to be highly resistant, exhibiting transient biphasic proliferation and recovery of viability even at high doses. A consolidated analysis established a clear hierarchy of sensitivity: TPC-1 > BCPAP > Nthy-ori3-1 > 8505C, revealing that papillary thyroid carcinoma cells are the most vulnerable. Crucially, a concentration of 65 µg/L, which is below current regulatory limits, triggered proliferative responses in all cell models.

**Discussion:**

These results suggest that Roundup® is a potent endocrine disruptor that exerts mutation-specific proliferative effects on thyroid cancer cells. Consistent growth stimulation at low, environmentally relevant doses suggests a potential tumor-promoting role, particularly in papillary carcinomas driven by the MAPK pathway. This evidence reinforces the need to revise risk assessments of GBHs (subcutaneous hypothyroidism) to take into account endocrine disruption and the potential for cancer progression beyond traditional toxicity.

## 1 Introduction

The global incidence of thyroid cancer has increased dramatically in recent decades, establishing it as one of the fastest-growing neoplasms (1). Although improved diagnostic sensitivity explains a substantial part of this increase, epidemiological evidence points to a concurrent role for environmental factors, given that the trend also encompasses larger tumors and an evolving histological and genetic landscape (2, 3). Mounting evidence implicates endocrine-disrupting chemicals (EDCs) as potential contributors to thyroid tumorigenesis, likely through their interference with hormonal signaling pathways critical for cellular homeostasis (4, 5).

Among the pervasive environmental contaminants, glyphosate-based herbicides (GBHs) are particularly concerning. The International Agency for Research on Cancer (IARC) has classified glyphosate as a Group 2A carcinogen, “probably carcinogenic to humans” (6). However, commercially available formulations such as Roundup^®^ can have effects that differ significantly from those of glyphosate alone, as proprietary adjuvants can enhance their bioavailability and toxicity (7, 8). This distinction is critically important, yet frequently overlooked in regulatory risk assessments, which predominantly rely on data from isolated active ingredients. This narrow focus is especially problematic in the context of EDCs, which are characterized by their ability to induce non-monotonic dose-response curves, where low doses can produce unpredictable, often paradoxical effects not observed at higher doses (9). This phenomenon fundamentally challenges traditional toxicological models based on monotonicity and underscores the necessity of testing whole-herbicide formulations at environmentally relevant concentrations.

The thyroid gland is highly vulnerable to these disruptive chemicals. EDCs impair thyroid hormone (T3 and T4) action, disrupt the hypothalamic-pituitary-thyroid axis, and are associated with hypothyroidism and goiter (10, 11). However, the direct effects of GBHs on thyroid cells, particularly neoplastic cells with diverse genetic backgrounds, are poorly understood. Our previous study demonstrated that Roundup^®^ Original DI exerts a dual, non-monotonic effect on human thyroid cells, inducing both toxicity and proliferation (12). Specifically, exposure to concentrations equivalent to the Brazilian Acceptable Occupational Exposure Level (AOEL, 160 µg/L) and Acceptable Daily Intake (ADI, 830 µg/L) caused significant cell death in normal follicular cells (Nthy-ori 3-1) and papillary thyroid carcinoma cells (TPC-1) after 24 h of exposure. Notably, this cytotoxic effect was attenuated after 48 h, and lower concentrations of the herbicide stimulated proliferation, an effect that was more pronounced in malignant TPC-1 cells (12). These findings suggest that thyroid cells, particularly cancerous ones, may activate adaptive survival mechanisms that enhance their proliferative potential after herbicide exposure.

Given the established heterogeneity of thyroid cancer, which manifests in subtypes with varying aggressiveness and therapeutic responses, it is crucial to determine whether these effects are consistent across various molecular contexts. Papillary thyroid carcinoma (PTC), the most common form, is frequently driven by mutations that activate the MAPK/ERK pathway, such as BRAF V600E mutation (13). In stark contrast, anaplastic thyroid carcinoma (ATC) is a rare but highly lethal malignancy characterized by aggressive behavior and resistance to conventional therapy (14).

Therefore, the present study builds upon our prior findings by comparing the effects of Roundup^®^ Original DI on two genetically distinct thyroid cancer cell lines: BCPAP, a papillary thyroid carcinoma (PTC) model derived from poorly differentiated thyroid carcinoma, positive for BRAF V600E, and 8505C, an ATC model that harbors p53 and other mutations (13). We hypothesized that the herbicide would elicit cell-type-dependent responses, reflecting the intrinsic genetic and metabolic disparities between differentiated carcinomas and lethal anaplastic tumors. By assessing cell viability, metabolic activity, and proliferation, this study aimed to elucidate how a common environmental pollutant may differentially influence thyroid cancer pathogenesis based on the molecular profile, thereby providing critical insights into its potential role in the progression of cancer.

## 2 Methodology

### 2.1 Cell culture and treatments

Two distinct thyroid carcinoma cell lines were used in this study: BCPAP (RRID: CVCL_0153), derived from papillary thyroid carcinoma and harboring the *BRAF* V600E mutation, and 8505C (RRID: CVCL_1054), derived from anaplastic thyroid carcinoma. Both cell lines were generously provided by Prof. Valdemar Máximo (Institute of Pathology and Molecular Immunology - Ipatimup, University of Porto, Portugal). The cell lines were cultures in RPMI 1640 medium supplemented with GlutaMAX™ and HEPES buffer (catalog #72400120, Gibco™), enriched with 10% fetal bovine serum (catalog #F9665, Sigma-Aldrich, St. Louis, USA), 1% penicillin-streptomycin solution (catalog #15140122, Gibco™), and 250 μg/mL fungizone (catalog #15290026, Gibco™) to prevent microbial contamination All cultures were maintained under standard conditions at 37 °C in a humidified atmosphere containing 5% CO_2_.

Cells were exposed to five distinct concentrations of Roundup^®^ Original DI (6.5, 65, 160, 830 and 6500 µg/L) for 24 and 48-hour periods, following previously established methodology (12). All experimental assays, including cell viability assessment via trypan blue exclusion, CCK-8 metabolic activity evaluation, and BrdU incorporation analysis, were performed in biological and technical triplicates (n = 3 independent experiments), including untreated control cells.

### 2.2 Statistical analysis

Statistical analyses were performed using the SAS System for Windows (version 9.4; SAS Institute Inc., Cary, NC, USA; 2002–2012). Descriptive statistics (mean, standard deviation, minimum, median, and maximum) were calculated to compare responses between concentrations for each cell line. The normality of the data was assessed using the Kolmogorov–Smirnov test. Since the data did not meet the assumptions of normal distribution, statistical analyses were performed using a three-way repeated-measures ANOVA (cell type × time × concentration) with rank transformation. Multiple comparisons, including comparisons between the present results and previously published data for Nthy-ori 3–1 and TPC-1 cell lines, were performed using Tukey’s *post hoc* test. The Wilcoxon test was additionally used for comparisons between BCPAP and 8505C when appropriate. A significance level of 5% (p < 0.05) was adopted for all analyses.

The results are presented as mean ± standard error (SE) in the main figures to facilitate visualization of experimental trends. However, complete descriptive statistics (median and interquartile range – IQR) for all experiments are provided in **Supplementary Tables 1**, **3**, **5**.

## 3 Results

Data represent mean ± SE from three independent experiments performed in triplicate. The results of the CCK-8 and BrdU assays were normalized to the control values ​​(set at 100%).

### 3.1 Effect of Roundup^®^ on cell viability

**Figure 1:**
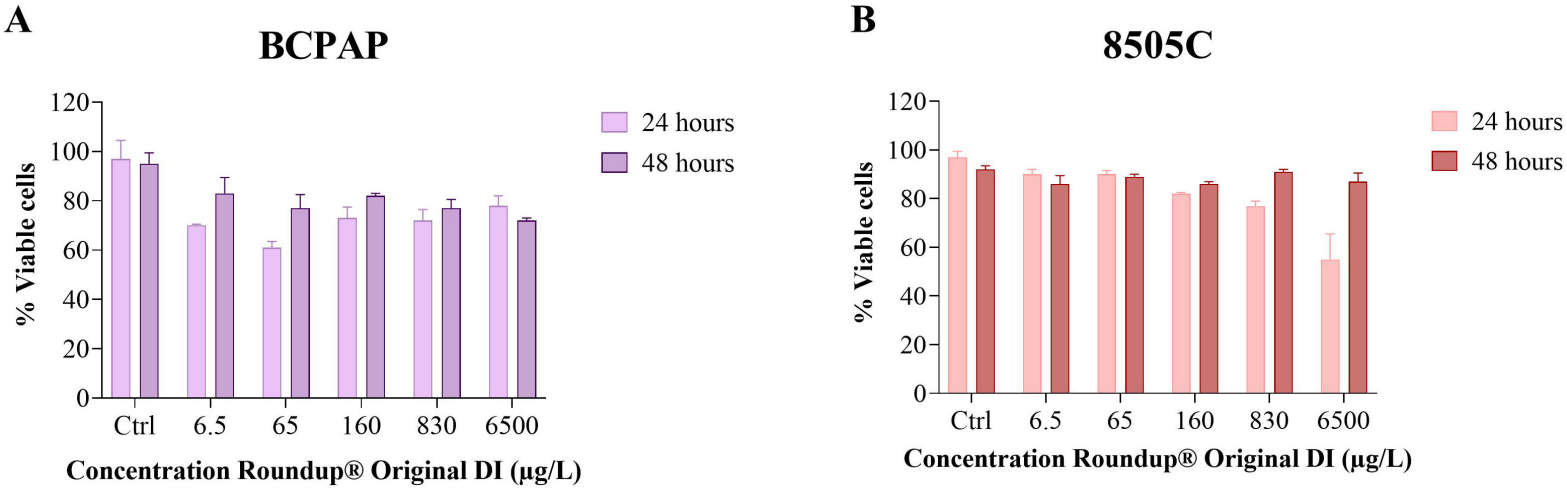
Cell viability of BCPAP **(A)** and 8505C **(B)** cell lines after exposure to Roundup^®^ Original DI for 24 h and 48 h, assessed by the Trypan Blue exclusion assay. Data represent mean ± SE, n = 3.

Both BCPAP and 8505C cell lines exhibited relative resistance to Roundup^®^ exposure, as measured using the trypan blue exclusion assay (**Figure 1**). This pattern aligns with the previously documented recovery of cell viability after 48 h in the Nthy-ori 3–1 and TPC-1 cell lines (12). The BCPAP cell line proved more sensitive, showing the most significant reductions in viability (61% and 70% after 24 h at 65 µg/L and 6.5 µg/L, respectively). At 48 h, the lowest viability (72%) was observed at the highest concentration (6500 µg/L). In contrast, the 8505C line was more resistant, with a significant reduction in viability (to 55%) occurring only at 6500 µg/L after 24 h; all other concentrations maintained viability above 70%. Notably, the 8505C line showed clear recovery by the 48-hour mark, with viability at the highest concentration increasing to 91% (**Table 1**).

**Table 1:** Percentage of viable BCPAP and 8505C cells after exposure to ^®^ Original DI for 24 h and 48 h, assessed using the Trypan Blue exclusion assay.

Concentration (µg/L)	BCPAP				8505C			
	24 hours	p value	48 hours	p value	24 hours	p value	48 hours	p value
Control	97% ± 7.5	–	95% ± 4.5	–	97% ± 2.5	–	92% ± 1.5	–
6.5	70% ± 0.5	**0.0006**	83% ± 6.5	0.0728	90% ± 2.0	0.3154	86% ± 3.5	0.1552
65	61% ± 2.5	**0.0005**	77% ± 5.5	**0.0074**	90% ± 1.5	**0.0032**	89% ± 1.0	**0.0359**
160	73% ± 4.5	**0.0353**	82% ± 1.0	**0.0377**	82% ± 0.5	**0.0034**	86% ± 1.0	**0.0098**
830	72% ± 4.5	**0.0311**	77% ± 3.5	**0.0025**	77% ± 2.0	**0.0352**	91% ± 1.0	**0.0428**
6500	78% ± 4.0	0.0695	72% ± 1.0	**0.0023**	55% ± 10.5	**0.0428**	87% ± 3.5	0.2114

Values are expressed as mean ± SE, n = 3. The p value refers to the comparison between the number of viable cells exposed to the herbicide and the number of viable control non-exposed cells. Bold values indicate statistically significant p-values for comparisons between each concentration and the control group.

**Figure 2:**
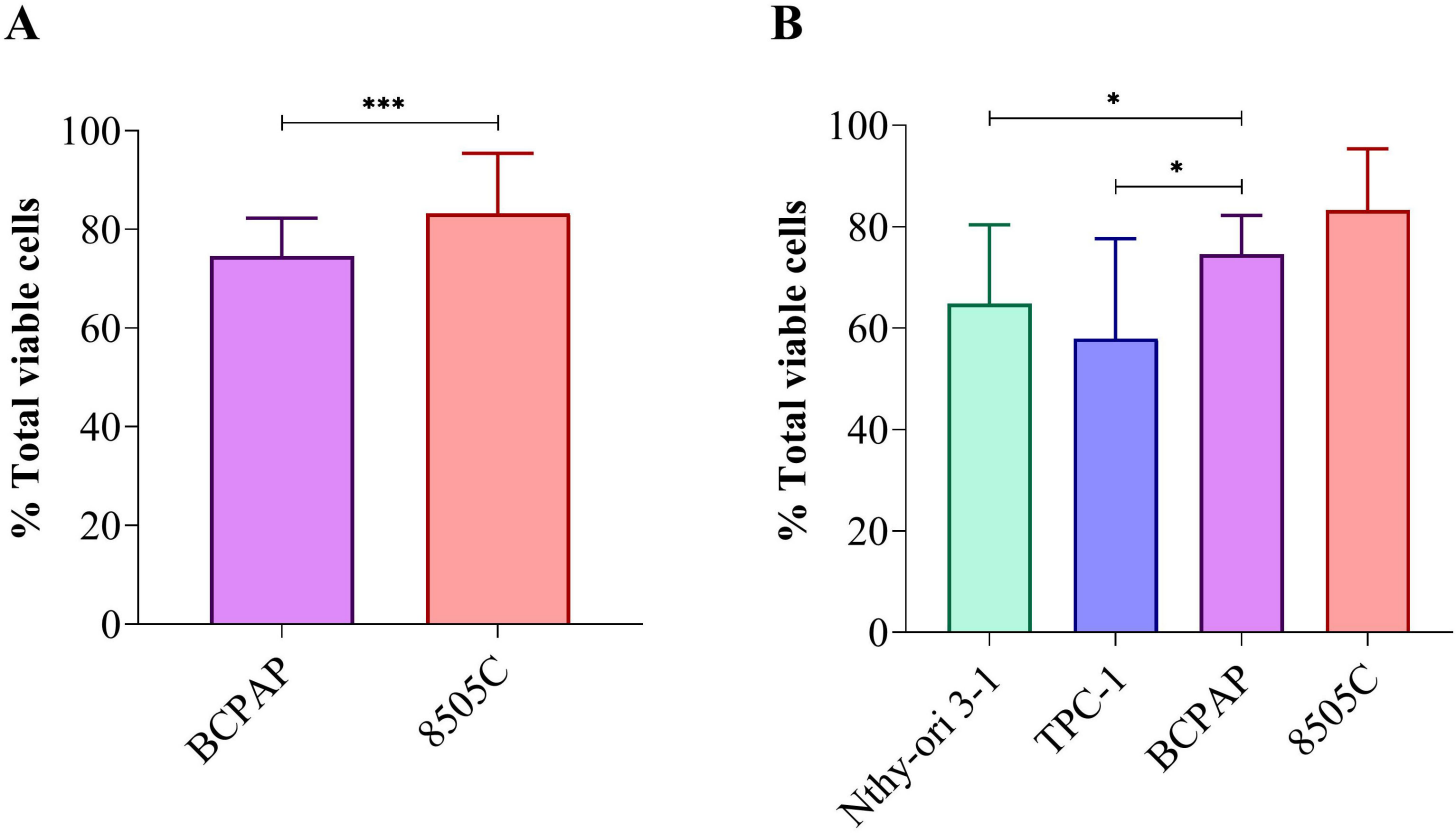
Comparison of cell viability after exposure to Roundup^®^ Original DI at different concentrations (control, 6.5, 65, 160 and 830 µg/L) for 24 h and 48 h: **(A)** Wilcoxon test between BCPAP and 8505C thyroid carcinoma cell lines; **(B)** comparative analysis among four thyroid cell lines using ANOVA with Tukey’s *post hoc* test. Statistical significance p < 0.05 (*), p < 0.01 (**), p < 0.001 (***).

Wilcoxon test analysis revealed a significant difference in cell viability between the BCPAP and 8505C lines across all tested concentrations (p = 0.0007, **Figure 2A**).

When these findings were compared with previously reported data for Nthy-ori 3–1 and TPC-1 cell lines (12), analysis of variance (ANOVA) with Tukey’s *post-hoc* test demonstrated highly significant differences in Roundup^®^ sensitivity among all four thyroid cell lines (p < 0.0001). The sensitivity hierarchy revealed that TPC-1 was the most responsive to herbicide exposure, followed by Nthy-ori 3-1 (**Figure 2B**; **Supplementary Table 2**). Detailed descriptive statistics, including median and IQR, for all concentrations and time points in the four thyroid cell lines are provided in **Supplementary Table 1**.

### 3.2 Cytotoxicity assessment

**Figure 3:**
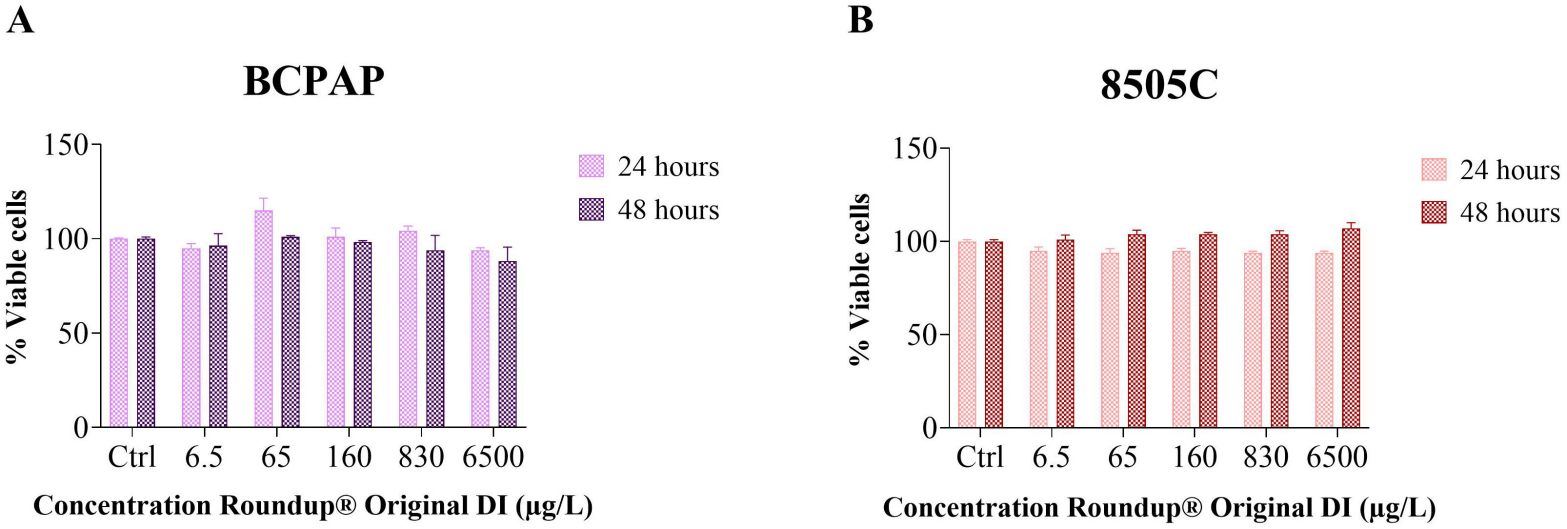
Cytotoxic effect of Roundup^®^ Original DI assessed by the CCK-8 assay in BCPAP **(A)** and 8505C **(B)** cell lines after 24 h and 48 h of exposure. Data represent mean ± SE, n = 3. Statistical comparisons were performed between each treated group and the respective control, and the *p* values are shown in **Table 2**.

The CCK-8 assay (**Figure 3**), which measures NAD(P)H-dependent metabolic activity, corroborated the trypan blue exclusion assay results, demonstrating sustained high cell viability across all tested concentrations and exposure times. These findings indicate that Roundup^®^ Original DI did not induce significant cytotoxicity in any of the thyroid cell lines tested.

Notably, several cell lines showed proliferative responses at specific concentrations. The Nthy-ori 3–1 cell line showed increased proliferation of 13% at 6.5 μg/L and 4% at 65 μg/L, while TPC-1 exhibited a 5% increase at 6.5 μg/L. The BCPAP cell line showed enhanced proliferation of 15% at 65 μg/L and 4% at 830 μg/L. In contrast, the 8505C cell line showed a delayed response, with proliferative increases (4% at 65, 160, and 830 μg/L, and 7% at 6500 μg/L) observed only after 48 hours of exposure (**Table 2**).

**Table 2:** Percentage of viable BCPAP and 8505C cells after exposure to Roundup^®^ Original DI for 24 h and 48 h, assessed by the CCK-8 assay.

Concentration (µg/L)	BCPAP				8505C			
	24 hours	p value	48 hours	p value	24 hours	p value	48 hours	p value
6.5	95% ± 5	0.4557	96.3% ± 12.9	0.2132	95% ± 4.2	0.3555	**101%** ± 4.7	0.6885
65	**115%** ± 13.1	**0.0202**	**101%** ± 0.7	0.2132	94% ± 4.3	0.4589	**104%** ± 3.5	0.5286
160	**101%** ± 9.5	0.6667	98,3% ± 1.5	0.4010	95% ± 2.6	0.1663	**104%** ± 1.6	0.8928
830	**104.3%** ± 5	0.3727	94% ± 15.7	0.1660	94% ± 1.5	0.3243	**104%** ± 3.5	0.7982
6500	94% ± 2.6	0.3701	88.3% ± 14.6	0.1204	94% ± 1.5	0.1192	**107%** ± 6.2	1.0000

Values are mean ± SE, n = 3. The highlighted values indicate increased proliferation relative to that of the control (set at 100%). The p value refers to the comparison between the number of viable cells exposed to the herbicide and the number of viable control nonexposed cells. Bold values indicate increased proliferation relative to the control (set at 100%). The p-value refers to the comparison between the number of viable cells exposed to the herbicide and the number of viable non-exposed control cells.

**Figure 4:**
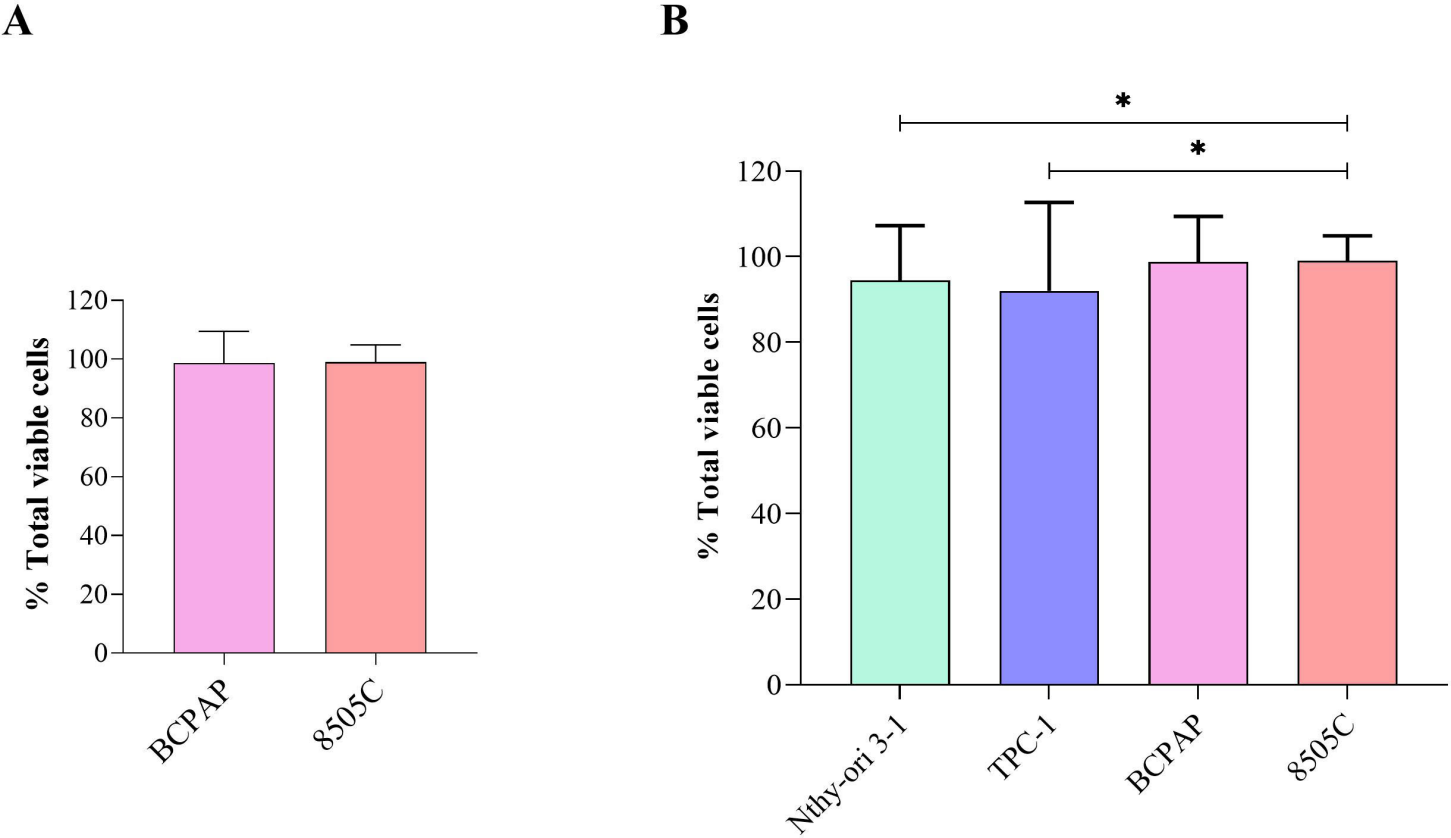
CCK-8 assay comparisons after exposure to Roundup^®^ Original DI at different concentrations (control, 6.5, 65, 160 and 830 µg/L) for 24 h and 48 h: **(A)** Wilcoxon test between BCPAP and 8505C thyroid carcinoma cell lines; **(B)** Comparative analysis between four thyroid cell lines using ANOVA with Tukey’s *post hoc* test. Statistical significance: p < 0.05 (*), p < 0.01 (**), p < 0.001 (***).

Although the concentrations tested did not show significant differences in relation to the control (untreated cells), the comparison between the cell lines, disregarding the control, revealed a significant difference by the ANOVA test for repeated measures (p = 0.0168; **Figure 4**; **Supplementary Table 4**). The complete absorbance values, expressed as median and IQR, for all experimental conditions are shown in **Supplementary Table 3**.

### 3.3 Influence of Roundup^®^ on cell proliferation

**Figure 5:**
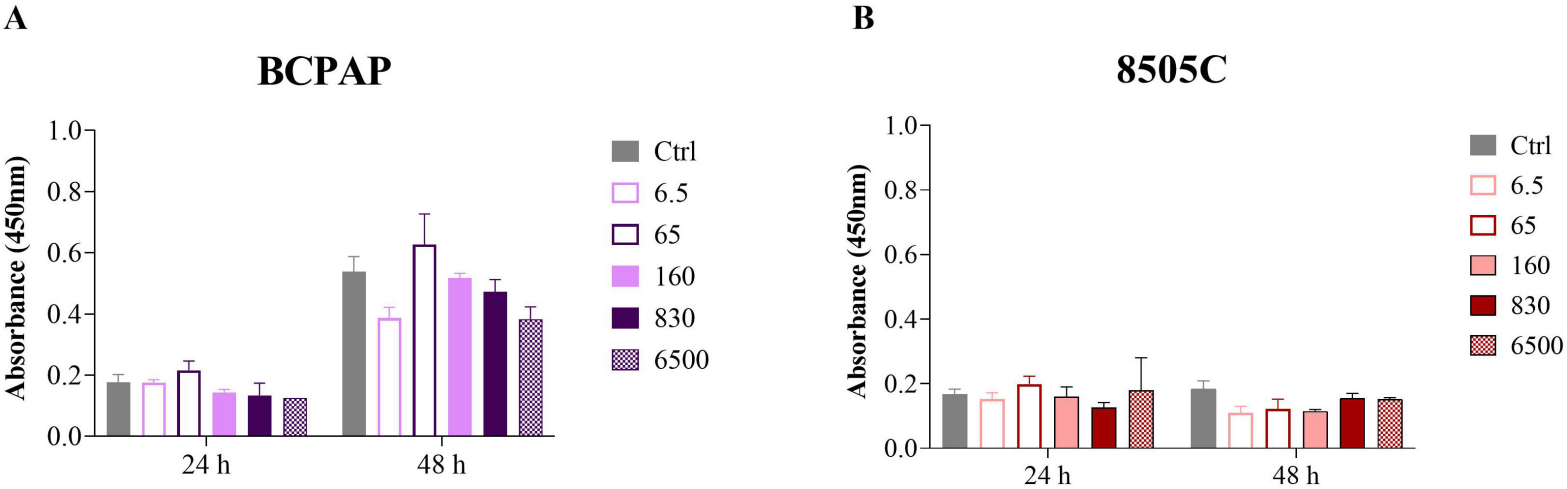
Cell proliferation in BCPAP **(A)** and 8505C **(B)** cell lines after exposure to Roundup^®^ Original DI for 24 h and 48 h. Proliferation was measured by BrdU incorporation, corrected for blank absorbance. Data are presented as mean ± SE from three independent experiments (n=3).

The BrdU incorporation assay supported the CCK-8 findings, indicating a trend toward increased proliferation at specific concentrations. BCPAP cells showed a slight increase in proliferation at 65 μg/L at both exposure times; however, this effect did not reach statistical significance compared to the control group. Similarly, the 8505C cell line showed a modest increase in proliferation at 65 μg/L and 6500 μg/L after 24 h, followed by reduced proliferation at several concentrations after 48 h (**Figure 5**, **Table 3**). Overall, no statistically significant differences in BrdU incorporation were observed between the treated cells and the control.

**Table 3:** Absorbance and percentage values for cell proliferation obtained by the BrdU assay in BCPAP and 8505C cells after exposure to Roundup^®^ Original DI for 24 h and 48 h.

Concentration (µg/L)	BCPAP						8505C					
	24 hours			48 hours			24 hours			48 hours		
	ABS	%	p value	ABS	%	p value	ABS	%	p value	ABS	%	p value
Control	0.177 ± 0.025	100	–	0.538 ± 0.050	100	–	0.168 ± 0.015	100	–	0.184 ± 0.025	100	–
6.5	0.175 ± 0.010	99	0.8780	0.387 ± 0.035	72	0.1252	0.152 ± 0.020	91	0.6737	0.110 ± 0.020	60	0.3193
65	0.216 ± 0.030	**122**	0.4482	0.627 ± 0.100	**117**	0.6734	0.198 ± 0.025	**118**	0.1145	0.122 ± 0.030	66	**0.0023**
160	0.143 ± 0.010	81	0.3184	0.518 ± 0.015	96	0.8635	0.160 ± 0.030	95	0.3474	0.115 ± 0.005	63	0.1175
830	0.134 ± 0.040	76	0.5813	0.473 ± 0.040	88	0.1917	0.127 ± 0.015	76	0.3078	0.115 ± 0.015	84	0.2490
6500	0.126 ± 0.000	71	0.2623	0.383 ± 0.040	71	0.1939	0.180 ± 0.100	**107**	0.6509	0.152 ± 0.005	83	0.5795

Values are expressed as mean ± SE (n = 3). Highlighted values indicate increased proliferation relative to the control (set as 100%). The p value refers to the comparison between the number of viable cells exposed to the herbicide and the number of viable control nonexposed cells. Bold values indicate increased proliferation relative to the control (set at 100%). The p-value refers to the comparison between the number of viable cells exposed to the herbicide and the number of viable non-exposed control cells.

**Figure 6:**
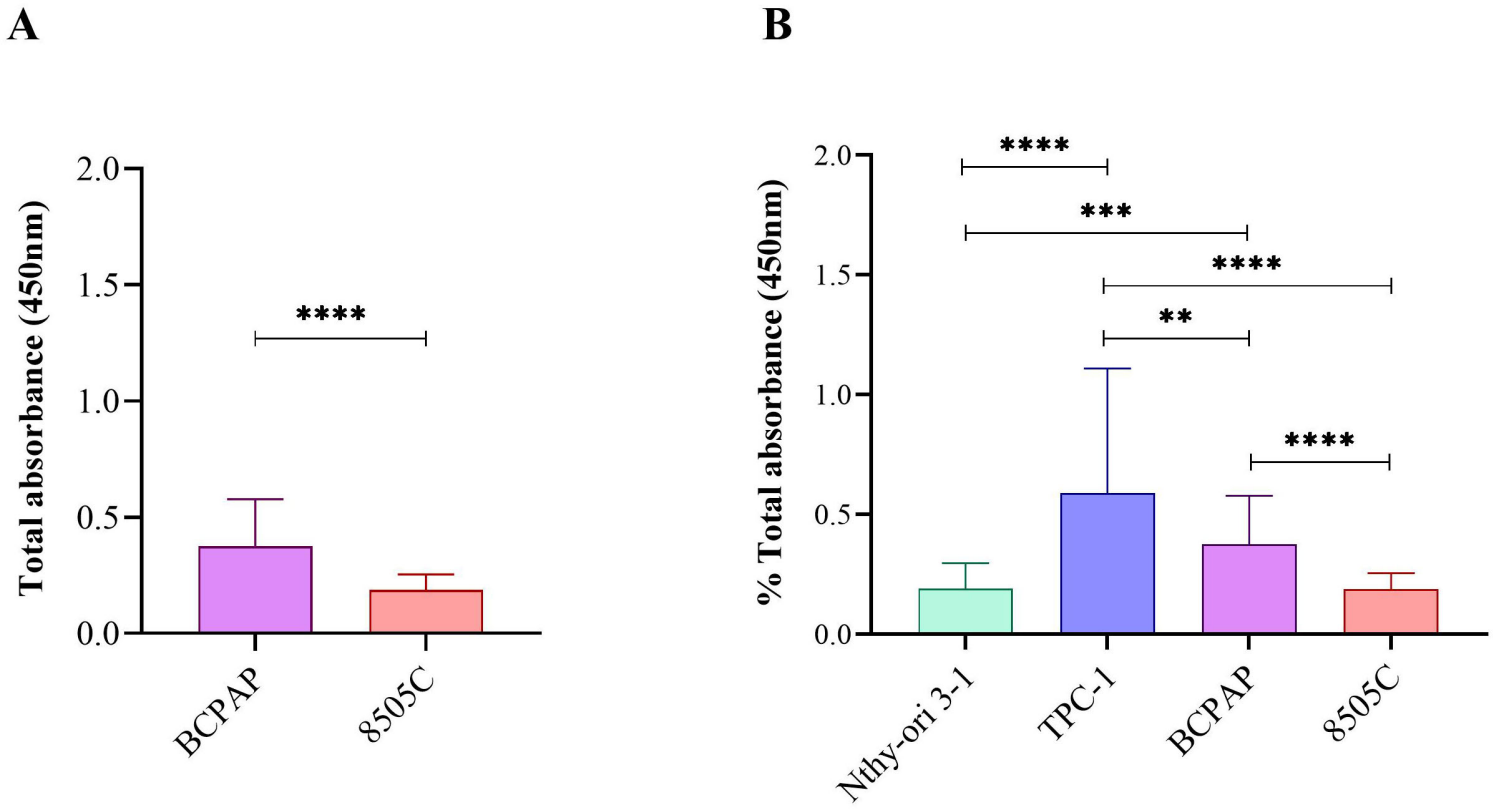
Comparison of cell proliferation after exposure to Roundup^®^ Original DI at different concentrations (control, 6.5, 65, 160 and 830 µg/L) for 24 h and 48 h: **(A)** analysis by Wilcoxon test between the BCPAP and 8505C lines; **(B)** comparative analysis among four cell lines using ANOVA with Tukey’s *post hoc* test. Statistical significance: p < 0.05 (*), p < 0.01 (**), p < 0.001 (***).

Statistical analysis using the Wilcoxon test revealed significantly higher proliferation in BCPAP compared to 8505C across all tested concentrations (p < 0.0001) (**Figure 6A**). Comparative analysis among all four thyroid cell lines using ANOVA followed by Tukey’s *post hoc* test revealed a proliferative hierarchy, with TPC-1 showing the highest proliferative response, followed by BCPAP (**Figure 6B**; **Supplementary Table 6**). Detailed results for all concentrations, exposure times, and cell lines, expressed as medians and IQR, are presented in **Supplementary Table 5**.

## 4 Discussion

This study revealed a complex, lineage-dependent disruptive effect of Roundup^®^ on thyroid cancer cells, characterized by a striking dissociation between cell viability and proliferation. While the anaplastic 8505C line demonstrated remarkable viability resistance, even recovering at high doses, papillary carcinoma derived BCPAP cells (BRAF V600E) were significantly more sensitive to treatment compared to 8505C. Notably, the herbicide did not induce cytotoxicity but elicited non-monotonic, endocrine-disruptive effects on metabolism and proliferation. Specifically, BCPAP cells exhibited sustained proliferation at a low concentration, whereas 8505C cells showed a transient biphasic response. Statistical analyses confirmed that papillary carcinoma cell lines were universally more proliferative and sensitive than their anaplastic or normal counterparts, establishing a clear hierarchy of susceptibility (TPC-1 > BCPAP > Nthy-ori 3-1 > 8505C). These findings suggest that Roundup^®^ Original DI may act as a tumor-specific mitogen, with significant implications for its role in the pathogenesis and progression of differentiated thyroid cancers.

The observed recovery of viability in the 8505C line after 48 h of exposure suggests the activation of robust adaptive and cellular repair mechanisms. This phenomenon aligns with the known metabolic plasticity and enhanced stress tolerance of highly aggressive cancer cell lines, which possess a greater capacity to withstand environmental insults (15–18). The sustained high metabolic activity measured by the CCK-8 assay across all cell lines corroborates that the primary effect of Roundup^®^ is not cytotoxicity but rather modulation of cell fate towards either death or proliferation, depending on the cellular context (19, 20).

The most significant finding of this study was the consistent proliferative stimulus induced by low concentrations of Roundup^®^ Original DI, particularly in papillary carcinoma cells. This paradoxical effect, where a toxic agent stimulates cell growth at low doses, is a hallmark of non-monotonic responses typical of endocrine disruptors (9, 21). The BCPAP and TPC-1 cell lines, which harbor mutations that activate the MAPK/ERK signaling pathway (RET/PTC and BRAF V600E, respectively), appear particularly susceptible to this proliferative trigger (13, 22–24). This suggests that pre-existing oncogenic signaling may synergize with the herbicide’s disruptive action, potentially creating a permissive environment for tumor-cell expansion. The fact that a concentration of 65 µg/L elicited responses in all four tested cell lines is critically important, as it indicates that biologically relevant effects occur at doses lower than the currently regulated AOEL (160 µg/L) and ADI (830 µg/L) levels established by regulatory agencies, such as the Brazilian ANVISA (25). Interestingly, in BCPAP cells treated with 65 µg/L for 24 h, the trypan blue assay indicated a reduction in cell viability, whereas the CCK-8 and BrdU assays did not show a decrease in metabolic activity or proliferation at the same time point. This difference can be explained by the fact that these assays measure different biological parameters. The trypan blue exclusion method evaluates membrane integrity and may underestimate early cellular responses, whereas CCK-8 reflects metabolic activity, and BrdU incorporation directly measures DNA synthesis and cell proliferation. Thus, cells under early stress conditions may show reduced viability in dye exclusion assays while still maintaining metabolic activity or proliferative capacity. Similar differences between membrane integrity and metabolic/proliferation assays have been widely reported (26–28).

Human biomonitoring studies have reported measurable levels of glyphosate in the urine of both the general population and occupationally exposed individuals. Concentrations reported in the general population range from 0.16 to 7.6 µg/L, whereas higher levels (up to 73.5 µg/L) have been detected in occupationally exposed individuals (29). Furthermore, recent biomonitoring studies in children living in agricultural areas have reported average concentrations of 7.43 µg/L, with values ​​reaching 38.3 µg/L (30). These data indicate that the concentrations tested in the present study, particularly 6.5 µg/L and 65 µg/L, are within or close to the range already detected in human biological matrices.

Our findings contribute to the growing mechanistic understanding of how GBHs influence thyroid cancer pathogenesis. We propose that GBHs do not necessarily initiate tumorigenesis *de novo* but may act as promoters, stimulating the proliferation of already transformed follicular cells. This hypothesis has substantial public health implications. Given the high prevalence of thyroid nodules in the general population, many of which harbor subclinical mutations (31, 32), widespread exposure to low levels of GBHs may provide a selective proliferative advantage to these mutated clones. This mechanism could contribute to the observed global increase in thyroid cancer incidence, not by increasing the number of new tumors, but by promoting the progression of pre-existing, often indolent microcarcinomas to clinically significant disease (33, 34). Our data provide a plausible biological pathway linking common environmental exposure to the tumor progression arm of thyroid cancer epidemiology.

This study had several limitations that should be acknowledged. First, although controlled, the *in vitro* model cannot fully recapitulate the complex hormonal and stromal interactions of the human thyroid gland. Second, the findings are specific to the Roundup^®^ Original DI formulation; other GBHs with different adjuvant compositions may yield different results (7, 8). Third, our experiments assessed acute exposure (up to 48 h), and the effects of chronic, low-dose exposure, which more accurately mirrors human environmental contact, remain unknown. Finally, although we identified a proliferative effect, the precise molecular mechanisms, whether through direct hormonal receptor interactions, induction of oxidative stress, or modulation of signaling pathways, require further investigation.

In conclusion, our findings demonstrate that the Roundup^®^ formulation induces non-monotonic effects on cell viability and proliferation in thyroid cells, with responses strongly dependent on the cell lineage and oncogenic context. The increased susceptibility of papillary carcinoma cells, particularly those with activated MAPK signaling, suggests that the herbicide may function as a tumor promoter, providing a selective growth advantage to pre-existing mutated cell clones. Given that these effects occurred at environmentally relevant concentrations below current regulatory limits, our findings highlight the need to reassess safety guidelines. Taken together, our results support the hypothesis that glyphosate-based herbicides may contribute to tumor promotion through non-monotonic and cell type-specific effects. Further studies investigating the molecular mechanisms underlying chronic exposure to low doses of these compounds are essential to elucidate their potential role in the development of thyroid cancer.

## Data Availability

The raw data supporting the conclusions of this article will be made available by the authors, without undue reservation.

## Glossary

ADI: Acceptable Daily Intake
AOEL: Acceptable Occupational Exposure Level
ATC: anaplastic thyroid carcinoma
PTC: Papillary thyroid carcinoma
CCK-8: Cell Counting Kit – 8
EDCs: Endocrine-disruptors chemicals
GBH: Glyphosate-based herbicides
IARC: International Agency for Research on Cancer
ANVISA: National Health Surveillance Agency
T4: Thyroxine
T3: Triiodothyronine.
