# Supplementary material for: Roundup® Original DI promotes proliferation in papillary thyroid carcinoma cells: a comparative study highlighting cell-type-dependent effects
Source: Front Endocrinol (Lausanne). 2026 Apr 23;17:1745247. doi: 10.3389/fendo.2026.1745247 (PMC13149085; doi:10.3389/fendo.2026.1745247)
Supplement: Supplementary file 1 [file DataSheet1.pdf]

frontiers

Supplementary Material

Supplementary table 1. Trypan Blue viability assay in thyroid cell lines exposed to Roundup®. Cell viability (%) in BCPAP, 8505C, TPC-1, and Nthy-ori 3-1 cells after 24 and 48 h of exposure to Roundup® (6.5–6500 µg/L). Data are shown as median (Q1–Q3) from independent experiments. Control = untreated cells.

Concentration	BCPAP		8505C		TPC-1		Nthy-ori 3-1	
(µg/L)	24 hours	48 hours	24 hours	48 hours	24 hours	48 hours	24 hours	48 hours
Control	97 (96.0-97.5)	95 (94.5-97.0)	95 (93.5-96.5)	96 (95.5-97.5)	69 (67.0-73.0)	80 (76.0-85.0)	78 (76.0-82.5)	81 (80.0-82.0)
6.5	70 (69.5-70.0)	81 (80.5-84.5)	90 (88.0-92.0)	83 (81.5-88.5)	44 (37.0-53.5)	79 (73.0-83.5)	52 (50.0-52.5)	81(65.0-86.0)
65	62 (58,5-63.5)	78 (76.0-78.5)	90 (88.5-91.0)	90 (88.5-90.0)	38 (29.5-45.0)	65 (64.5-72.0)	65 (60.0-66.0)	67 (61.5-71.5)
160	77 (70.0-78.0)	82 (80.0-84.0)	82 (81.5-82.0)	85 (85.0-87.0)	43 (39.0-45.5)	68 (63.0-71.5)	55 (48.5-64.5)	76 (56.5-81.5)
830	77 (69.5-77.5)	77 (76.0-78.5)	78 (75.5-79.0)	90 (89.5-91.5)	42 (39.0-45.5)	71 (52.5-77.0)	63 (57.5-72.0)	85 (79.5-85.5)
6500	78 (74.5-82.0)	72 (71.5-72.5)	54 (44.5-65.0)	86 (83.5-90.5)	36 (33.5-49.0)	84 (83.0-86.0)	49 (45.0-50.0)	75 (73.0-79.5)

Supplementary table 2. ANOVA results for repeated measures compared by Trypan Blue Exclusion Assay (% viability cells*) between cell types, concentrations and times (n=3).

Cells	Concentrations (µg/L)	Times	Interactions			
Nthy-ori 3-1, TPC-1, BCPAP and 8505C	6.5, 65, 160, 830, 6500	24h and 48h	Concentrations*cell	Times*cells	Concentrations*times	Concentrations*times*cell
**<0.0001[a]**	**<0.0001**	**0.0151**	**0.0324**	0.2530	**0.0171**	**0.0061**

*Variable transformed into ranks for analysis.

[a] Significant effect for lineage type (BCPAP × Nthy-ori 3-1: p = 0.0404 and BCPAP × TPC-1: p = 0.0338).

Considering that the interaction between the three factors (time, concentration, and cell) is significant, we performed the following analyses.

Cell fixation and comparison of time and concentration

Supplementary table 2.1. Repeated-measures ANOVA for each cell.

	8505C	BCPAP	Nthy-ori 3-1	TPC-1
**Time**	p = 0.5362	p = 0.9625	p = **0.0313**	p = 0.1190
**Concentration**	p = **0.0020**	p = **0.0009**	p = 0.3327	p = **0.0150**
**Time*concentration**	p = **0.0064**	p = 0.1533	p = 0.1702	p = **0.0447**

Considering the significant interaction between the two factors (time and concentration), we perform the analysis.

Fixing time and comparing concentration in each cell type

Comparison between concentrations by cell type at 24h and 48h – **Only those that were significant**

Comparison of 24-hour concentrations by cell type:

8505C (6.5 × 830: p = 0.0011, 6.5 × 6500: p = 0.0182 and 65 × 160: p = 0.0112)
Nthy-ori 3-1 (65 × 6500: p = 0.0097)
TPC-1 (160 × 6500: p = 0.0359)

Comparison of 48-hour concentrations by cell type:

Nthy-ori 3-1 (65 × 830: p = 0.0486)
TPC-1 (65 × 6500: p = 0.0244)

Fixing concentration and comparing times for each cell type

Comparison between times (24h and 48h) for each concentration and cell type **– Only those that were significant**

8505C (830 → 24h × 48h: p = 0.0194)
BCPAP (6.5 → 24h × 48h: p = 0.0326 and 65 → 24h × 48h: p = 0.0058)
TPC-1 (65 → 24h × 48h: p = 0.0436 and 6500 → 24h × 48h: p = 0.0022)

Analysis of the concentration*cell type interaction

Comparison between cell types at each concentration – Only those that gave significant

6.5 µg/L (8505C × Nthy-ori 3-1: p = 0.0053 and 8505C × TPC-1: p = 0.0040)
65 µg/L (8505C × BCPAP: p = 0.0131, 8505C × Nthy-ori 3-1: p = 0.0048 and 8505C × TPC-1: p = 0.0008)
160 µg/L (8505C × Nthy-ori 3-1: p = 0.0018, 8505C × TPC-1: p = 0.0007, BCPAP × Nthy-ori 3-1: p = 0.0102 and BCPAP × TPC-1: p = 0.0033)
830 µg/L (8505C × TPC-1: p = 0.0019, BCPAP × TPC-1: p = 0.0262 and Nthy-ori 3-1 × TPC-1: p = 0.0415)

Supplementary Table 3. CCK-8 assay (absorbance values) in thyroid cell lines exposed to Roundup®. Absorbance values measured after 24 and 48 h of exposure to Roundup® (6.5–6500 µg/L) in BCPAP, 8505C, TPC-1, and Nthy-ori 3-1 cells. Data are presented as median (Q1–Q3) from independent experiments.

**Concentration**	**BCPAP**		**8505C**		**TPC-1**		**Nthy-ori 3-1**	
**(µg/L)**	**24 hours**	**48 hours**	**24 hours**	**48 hours**	**24 hours**	**48 hours**	**24 hours**	**48 hours**
6.5	2.0 (1.9-2.0)	2.5 (2.4-2.6)	2.7 (2.7-2.8)	2.7 (2.6-2.8)	2.5 (1.9-2.6)	2.3 (1.4-2.5)	1.0 (0.6-1.4)	2.6 (2.3-2.7)
65	2.1 (2.1-2.2)	2.6 (2.5-2.7)	2.8 (2.7-2.9)	2.6 (2.6-2.7)	2.3 (1.8-2.5)	2.3 (1.7-2.5)	1.4 (0.8-1.5)	2.3 (2.1-2.5)
160	1.9 (1.9-2.0)	2.7 (2.6-2.8)	2.8 (2.8-2.8)	2.6 (2.6-2.7)	1.9 (1.6-2.2)	2.2 (1.5-2.2)	0.9 (0.5-1.1)	2.4 (2.1-2.7)
830	2.0 (1.9-2.0)	2.6 (2.6-2.7)	2.7 (2.7-2.8)	2.7 (2.6-2.7)	2.2 (1.9-2.4)	2.3 (1.8-2.5)	0.8 (0.5-1.2)	2.4 (2.2-2.7)
6500	1.9 (1.9-2.0)	2.6 (2.4-2.6)	2.7 (2.7-2.8)	2.6 (2.6-2.6)	2.2 (1.9-22)	2.3 (1.9-2.3)	0.5 (0.8-1.2)	2.5 (2.1-2.8)

Supplementary table 4. ANOVA results for repeated measures compared by CCK-8 assay (% viability cells*) between cell types, concentrations and times (n=3).

Cells	Concentrations (µg/L)	Times	Interactions			
Nthy-ori 3-1, TPC-1, BCPAP and 8505C	6.5, 65, 160, 830, 6500	24h and 48h	Concentrations*cell	Times*cells	Concentrations*times	Concentrations*times*cell
**0.0168[a]**	**0.0005**	0.0973	**0.0005**	0.0865	0.6811	**0.0360**

*Variable transformed into ranks for analysis.
[a] Significant effect for lineage type (8505C × Nthy-ori 3-1: p = 0.0163 and 8505C × TPC-1: p = 0.0360).

Considering that the interaction between the three factors (time, concentration, and cell) is significant, we performed the following analyses.

Cell fixation and comparison of time and concentration

Supplementary table 4.1. Repeated-measures ANOVA for each cell.

	8505C	BCPAP	Nthy-ori 3-1	TPC-1
**Time**	p = 0.2564	p = 0.1248	p = 0.2071	p = 0.9764
**Concentration**	p = 0.2474	p = **0.0023**	p = **0.0290**	p = 0.5813
**Time*concentration**	p = 0.7234	p = 0.0851	p = 0.0637	p = 0.3597

Considering the significant interaction between the two factors (time and concentration), we perform the analysis.
Fixing time and comparing concentration in each cell type
Comparison between concentrations by cell type at 24h – **Only those that were significant**

Comparison of 24-hour concentrations by cell type:
8505C (6.5 × 830: p < 0.0001)
BCPAP (65 × 830: p = 0.0399)

Fixing concentration and comparing times for each cell type
Comparison between times (24h and 48h) for each concentration and cell type **– Only those that were significant**

8505C (160 → 24h × 48h: p = 0.0471 and 6500 → 24h × 48h: p = 0.0238)
Nthy-ori 3-1 (160 → 24h × 48h: p = 0.0373)

Supplementary Table 5. Absorbance values obtained in the BrdU incorporation assay in four thyroid cell lines (BCPAP, 8505C, TPC-1, and Nthy-ori 3-1) after 24 and 48 hours of exposure to increasing concentrations of Roundup® (6.5–6500 µg/L). Results are presented as **median (interquartile range: Q1–Q3)** from independent experiments.

Concentration (µg/L)	BCPAP		8505C		TPC-1		Nthy-ori 3-1	
	24 hours	48 hours	24 hours	48 hours	24 hours	48 hours	24 hours	48 hours
Control	0.2 (0.2-0.2)	0.5 (0.5-0.6)	0.2 (0.2-0.2)	0.2 (0.2-0.2)	0.5 (0.5-0.5)	0.3 (0.1-0.1)	0.2 (0.2-0.2)	0.1 (0.1-0.1)
6.5	0.2 (0.2-0.2)	0.4 (0.3-0.4)	0.1 (0.1-0.2)	0.1 (0.1-0.1)	0.5 (0.6-1.6)	0.4 (0.3-0.4)	0.1 (0.1-0.1)	0.3 (0.3-0.4)
65	0.2 (0.2-0.2)	0.5 (0.5-0.7)	0.2 (0.2-0.2)	0.2 (0.1-0.2)	0.5 (1.0-1.5)	0.3 (0.2-0.2)	0.2 (0.2-0.3)	0.2 (0.1-0.3)
160	0.1 (0.1-0.2)	0.5 (0.5-0.5)	0.1 (0.1-0.2)	0.1 (0.1-0.1)	0.5 (0.5-1.2)	0.4 (0.2-0.2)	0.1 (0.1-0.2)	0.2 (0.2-0.2)
830	0.1 (0.1-0.2)	0.4 (0.4-0.5)	0.1 (0.1-0.1)	0.2 (0.1-0.2)	0.5 (0.5-0.6)	0.3 (0.2-0.3)	0.2 (0.1-0.2)	0.2 (0.1-0.2)
6500	0.1 (0.1-0.1)	0.4 (0.4-0.4)	0.1 (0.1-0.2)	0.2 (0.1-0.2)	0.5 (0.5-0.7)	0.3 (0.3-0.7)	0.1 (0.1-0.1)	0.1 (0.1-0.3)

Supplementary table 6. ANOVA results for repeated measures comparing BrdU assay (cell proliferation absorbance*) between cell types, concentrations and times (n=3).

**Cells**	**Concentrations (µg/L)**	**Times**	**Interactions**			
Nthy-ori 3-1, TPC-1, BCPAP and 8505C	6.5, 65, 160, 830, 6500	24h and 48h	Concentrations*cell	Times*cells	Concentrations*times	Concentrations*times*cell
**<0.0001[a]**	0.1547	**0.0040**	**0.0004**	**0.0312**	0.3509	0.8814

*Variable transformed into ranks for analysis.

[a] Significant effect for lineage type (8505C x BCPAP: p= <0.0001; 8505C x TPC-1: p= <0.0001; BCPAP x Nthy-ori 3-1: p= 0.0009; BCPAP x TPC-1: p= 0.0077 and Nthy-ori 3-1 x TPC-1: p= <0.0001).
